# Transparency of peer review: a semi-structured interview study with chief editors from social sciences and humanities

**DOI:** 10.1186/s41073-021-00116-4

**Published:** 2021-11-18

**Authors:** Veli-Matti Karhulahti, Hans-Joachim Backe

**Affiliations:** 1 Faculty of Humanities and Social Sciences, University of Jyväskylä, Jyväskylä, Finland; 2 Department of Digital Design, IT University of Copenhagen, Copenhagen, Denmark

**Keywords:** Ethics, Journalology, Open Science, Peer review, Social sciences and humanities

## Abstract

**Background:**

Open peer review practices are increasing in medicine and life sciences, but in social sciences and humanities (SSH) they are still rare. We aimed to map out how editors of respected SSH journals perceive open peer review, how they balance policy, ethics, and pragmatism in the review processes they oversee, and how they view their own power in the process.

**Methods:**

We conducted 12 pre-registered semi-structured interviews with editors of respected SSH journals. Interviews consisted of 21 questions and lasted an average of 67 min. Interviews were transcribed, descriptively coded, and organized into code families.

**Results:**

SSH editors saw anonymized peer review benefits to outweigh those of open peer review. They considered anonymized peer review the “gold standard” that authors and editors are expected to follow to respect institutional policies; moreover, anonymized review was also perceived as ethically superior due to the protection it provides, and more pragmatic due to eased seeking of reviewers. Finally, editors acknowledged their power in the publication process and reported strategies for keeping their work as unbiased as possible.

**Conclusions:**

Editors of SSH journals preferred the benefits of anonymized peer review over open peer and acknowledged the power they hold in the publication process during which authors are almost completely disclosed to editorial bodies. We recommend journals to communicate the transparency elements of their manuscript review processes by listing all bodies who contributed to the decision on every review stage.

**Supplementary Information:**

The online version contains supplementary material available at 10.1186/s41073-021-00116-4.

## Introduction

A recent cross-disciplinary review of scientific journals’ instructions found Social Sciences and Humanities (SSH) journals disclosed their peer review practices more than other disciplines: humanities – 61%, social sciences – 72%, while physical sciences – 41% [1]. Another study in 2019 showed that 174 journals were using open peer review, but only one (1%) of those were from the humanities [2]. As the debate about advantages and disadvantages of open peer review continues today especially in medical sciences [3, 4], very little is known about peer review transparency in SSH, which is the topic of the present study.

In the above literature, “open peer review” has generally two meanings: peer review in which authors and/or reviewers are disclosed to each other, and the public sharing of peer review reports. A recent taxonomy [5] has suggested a third level of potential openness: transparency related to communications between authors, editors, and reviewers (see also Supplement). In this article, we discuss all three domains of peer review “openness,” but unless otherwise noted, we use the term “open peer review” by the first category: authors, editors, and/or reviewers being disclosed to each other during the various stages of review. In fully open peer review, all identities are disclosed to all parties.

Tennant and Ross-Hellauer’s comprehensive mapping of the state of peer review research [6] identifies several unstudied research questions in the field – e.g., the justifications for editorial decisions and the epistemic diversity of peer review – which relate to disciplinary differences and, arguably, the SSH in particular. In the same way as the increasing demands for data sharing have resulted in numerous SSH-driven debates due the special epistemological issues in the qualitative domain [7, 8], there are good reasons to expect SSH to evolve also with different peer review standards compared to, for instance, medical and natural sciences.

Related to the above, Guetzkow and colleagues [9] conducted an interview study with peer reviewers from diverse fields and found natural sciences scholars to conceive of originality very narrowly as the “production of new findings and theories,” whereas for SSH peer reviewers originality could also be associated with novel elements in the approach, method, data, topic, or simply the fact that the research was linked to an understudied area. Considering that some originality statements might be less easy to justify than others, such conceptual differences may also resonate with the degree to which authors, editors, and reviewers are willing to disclose their identities to others during review processes. Similar differences can be found in other key concepts, such as replicability [10, 11], which further supports the need for disciplinary-specific investigations.

Although megajournals, like *PLOS ONE*, which welcome manuscripts from virtually all disciplines, are changing the peer review landscape both through publication processes and outside of them (e.g., via assessments in funding bodies), most of the scientific dialogue still takes place in discipline-specific journals, including those identifying with SSH subfields. Accordingly, this interview study was set up to answer three research questions:

- RQ1: How do highly-ranked SSH journals perceive open peer review processes and do these perceptions materialize in the actual processes they employ?
- RQ2: How do editors in chief position their journals’ review and publication practice between policy, ethics, and pragmatism?
- RQ3: How do the editors in chief situate their own powerful role in the manuscript review process and how is that role negotiating open science principles of the review process?

### Further literature

Related to RQ1, Glonti and colleagues [12] recently carried out an interview study with biomedical journal editors and found that “each journal’s unique context and characteristics, including financial and human resources and journal reputation” (p. 7) affected editorial decision making and their expectations towards peer reviewers. This supports the premise that journals within SSH, being delineated by their disciplinary characteristics, may view and practice open peer review in distinct ways. The above contextualizes also our RQ2, as “financial and human resources” might play a pragmatic role in peer review transparency; for instance, finding peer reviewers who agree to write open reports can be resource-intensive [13].

A lot has been written about the ethics of peer review, which further relates to RQ2. For instance, one identified problem for both open and anonymized peer review formats is what Gorman has coined the “Oppenheim effect:” a known-author syndrome according to which some editors and peer reviewers (“We are going to publish it anyway”) tend to provide established scholars with special treatment [14]. Recently, this effect has received empirical support, as Tomkins and associates [15] had half a thousand papers peer reviewed by four experts, two of which saw the authors’ names and two had them anonymized. Peer reviewers were much more likely to accept papers from famous authors and top institutions, compared with their anonymized counterparts. The same study also found a statistically significant bias against women authors – one more issue that has been known to problematize peer review for decades [16], albeit recent findings indicate change in this regard [17].

As to our RQ3, Wager and colleagues [18] surveyed 231 editors in chief regarding publication ethics and found that most editors “appear not to be very concerned about publication ethics issues [and] feel reasonably confident that they can handle publication ethics issues” (p. 256). Lipworth’s team [19], in turn, interviewed biomedical journal editors regarding journal peer review. These editors generally perceived subjective influences in the review process positively and considered them as expressions of both the editors’ and reviewers’ epistemic authority and expertise.

Although we expand on the above existing findings, our study is not built on exact hypotheses. We did entertain and preregister some expected trends before data collection (https://osf.io/wjztp), but due to the nonconfirmatory nature of this study, these expectations will not be discussed in detail. Future quantitative studies with good statistical power will be more suitable for hypothesis testing.

## Method and materials

We answer our research questions by means of 12 semi-structured interviews with editors in chief of respected SSH academic journals. We use the term “respected” to indicate that our focus is on journals that are recognized as quality academic platforms (excluding so-called “predatory journals,” etc.). The study followed the Finnish National Board on Research Integrity guidelines, according to which this type of study was exempted from ethical review. Before reaching out for participants, we created an interview structure and work plan which were stored in the Open Science Framework on June 25, 2020 (https://osf.io/wjztp). The idea was that registering questions and general objectives of the research beforehand would build up both credibility as well as trust, and thus facilitate approaching busy editors in chief who presumably receive many contact requests daily. Further details such as the complete question list and outcome expectations were not disclosed but embargoed, as we did not want to influence the interviewees’ responses. After preregistration and testing, small changes were made to the original 21 questions (for final questions list see Supplement). All questions were asked to all interviewees, although we also posed follow-up questions when we did not understand the answers or when we received answers that in our opinion required further exploration.

### Participants

The sample size was not generated by saturation; rather, it was predefined by our estimation of *N* = 12 being sufficient to answer the research questions [20, 21]. We did not choose the journals based on metrics such as impact factor, albeit many of the interviewed journals did rank very high according to these figures (the average impact factor of the four journals that promoted it was 5.9). Two of our journals are not included at all among the 2021 impact factor rankings, but are at the very highest tier in both Danish and Finnish journal rankings (while one with a very high impact factor belonged to the lowest tier) [22]. Additionally, we considered it important to include journals with not only different scholarly domains, but different profiles and sizes: the smallest journal from the most narrowly defined field publishes only around 15 articles per year, whereas the three biggest journals all publish more than five times this volume.

A further goal was to balance the 12 interviews so that diverse journal geographical regions, subdisciplines, and editor genders would be represented. All interviewed editors in chief characterized their journal as multi- or interdisciplinary, even if their journal has a focus on or origin in a singular discipline. We created a list of 20 journals that we would approach by personal email one by one so that when our invitation would be ignored or rejected, a new journal with similar representation was selected. Two editors refused to be interviewed due to time constraints and four did not answer, but we had no trouble finding volunteers, as the rest of the editors were interested in discussing the topic and willing to make time in their schedules. An open call for participation was also distributed on Twitter, but we did not receive any responses.

Some journals had multiple editors in chief. One interview included two editors from the same journal and another interview had a second editor in chief replacing the other after initial communication. We do not disclose further details about the editors or their journals to protect their privacy. Many of the interviewees explicitly wanted to remain anonymous. In light of the above, our method can be described as purposive sampling. A summary of journal characteristics is presented in Table 1.

**Table 1:** Characteristics of the 12 Social Sciences and Humanities journals participating in this study

Journal	Field*	Publ./Year	Open access	Journal age	Editor tenure	N° of editors in chief
1	H	> 10	yes	> 20	> 20	1
2	H	> 10	no	> 20	> 20	1
3	SSH	> 100	no	> 20	> 20	1
4	SSH	> 20	no	> 50	time-limited	1
5	SS	> 50	no	> 40	< 5	< 5
6	SSH	> 20	no	> 30	> 30	> 5
7	SSH	> 20	yes	> 40	time-limited	< 5
8	SSH	> 200	yes	> 10	> 10	1
9	H	> 20	no	< 10	> 5	1
10	SSH	> 10	yes	> 30	< 5	1
11	H	> 20	no	< 10	> 5	< 5
12	SS	> 50	yes	< 10	> 5	1

* *H* humanities (literature, philosophy, etc.); *SS* social science (psychology, sociology, etc.); *SSH* mixed H and SS (e.g., communication and media)

### Data and analysis

The interviews were carried out in English using Zoom remote communication software from home offices (during the COVID-19 pandemic). Only the audio part of the video calls was stored for analysis, and their average recorded length was 67 min (*SD* = 8). A protected university cloud service was used for storing the data; every five years the need to continue keeping the stored data with be re-assessed. The participants were informed of the research details via email before the interview and in more detail at the beginning of the interview, during which the interviewees were also invited to ask questions related to the study. Informed consent was collected in digitally-signed PDF format. Both authors were present in all interviews, taking turns in asking the questions. For transparency, we state that both authors are senior researchers, identify as men, and three of the interviewees had been communicated with before at least by one of us. The first author had previous experience of conducting research interviews [23, 24].

The interviews were transcribed into text by using a General Data Protection Regulation-compliant service (Konch.ai). All texts were proofread with the help of an external assistant, as approximately 10% of the automated transcriptions were unreadable. The proofread text files were uploaded to a university-supported Atlas.ti system, which we used for software-based text analysis. To organize the data for use (and potential reuse), we decided to carry out open descriptive coding [25] instead of directly seeking answers to our research questions. The first author coded all text without a predesigned coding scheme (533 codes overall) and the second author inductively close read the data with marking (no coding software). Coding reliability was tested by having an external assistant unitize 17% of the data [26]; because the assistant did not have experience of academic peer review, we did not pursue high numeric interrater reliability but negotiated an agreement [27]. After merging overlapping codes and coding negotiation, 510 codes remained.

We did not pursue themes or a manual for new coding rounds, but reconciled differences via consensus [28]. Because both researchers participated in the development of the interview and actual interviews, agreement was reached in a single one-day session. The agreement formed 11 data domains, which are presented in Table 2.

**Table 2:** The data domains of the interviews with 12 Social Sciences and Humanities journals

Family	Content
**Authors**	comments related to the authors who submit to the journal.
**Decision**	comments related to manuscript publication decisions.
**Editing**	comments related to the work of journal editing in general.
**Editors**	comments related to the editor’s personal beliefs or role.
**Interview**	comments related to the interview in question.
**Journal**	comments related to the editor’s journal.
**Open science**	comments related to open science in general.
**Publisher**	comments related to the publisher of the editor’s journal.
**Review**	comments related to the review process in the editor’s journal.
**Reviewers**	comments related to external peer reviewers of the editor’s journal.
**Science**	other comments related to the scientific world and its developments.

The second author examined the data domains, to map sections as answers to our three research questions. Due to the thematic overlap in the data domains, many of them included codes that were relevant for multiple research questions; however, some research questions very strongly connected to some data domains.

- RQ1: editors, review, science.
- RQ2: authors, reviewers, publisher.
- RQ3: decision, editing, journal, open science.

The domain “interview” was only marginally relevant for our RQs, and its content will not be discussed in this article.

We will not systematically present the number of instances of all coded events. We acknowledge that a quantitative approach to the data can also be useful; however, in such cases the target journals should have been selected with narrower inclusion criteria for them to be representative (of a selected subfield that could be represented by 12 journals). Again, as the present goals are not confirmatory, we assert that our non-quantitative analysis of the data is more fitting. Due to the privacy concerns expressed by some interviewees, we do not share our data via an open repository. Parts of specific interest can be made available by request to the corresponding author.

Before publication, we applied member checking by allowing the interviewees to criticize and comment on the latest version of the manuscript. Two interviewees suggested minor revisions, which were included in the final publication.

## Results

We start with a summary and, below, findings regarding each RQ respectively. Overall, our interviews with the editors in chief of 12 respected SSH journals indicated a strong awareness of open peer review benefits, but overall preferences for anonymized review. This is best captured in the quote from one of the editors:

-
  “*I personally often think that many processes that take place in academia should be open and transparent … But I tend to be in the minority, and most of my colleagues disagree with me. So, I’m not sure, maybe, you know, maybe I’m being too naïve, or too optimistic about human nature.*” (E5)

It is important to note that all journals used double anonymized review, which was also widely considered to be the “gold standard” that authors and editors are expected to follow by default. In one exceptional case, both double anonymized and open reviews were used, depending on the submission.

The rationales for preferring double anonymized peer review were systematically related to the three areas of our second research question: policy, ethics, and practice. Anonymized peer review was cited, among others, as an institutional requirement that credible journals had to meet; moreover, the anonymized process was considered ethically superior by protecting both authors and reviewers, while also making the search for the latter easier (many reviewers refuse to sign their reports).

Following the double anonymous format, all editorial decisions were made with the authors’ names disclosed, and triple anonymous peer review – identities of decision makers would also remain hidden from authors and vice versa – was not considered (viable). The editors in chief hardly perceived this problematic, but either defended this as an important curatorial part of scientific publication (e.g., guaranteeing fair treatment for authors from all groups) or explained strategies that were applied to distribute their power, for instance, by basing the decisions on the opinions of multiple experts.

### How do highly ranked SSH journals perceive open peer review processes and do these perceptions materialize in the actual processes they employ?

The editors of the interviewed journals perceived open peer review mainly as a (weaker) alternative to anonymized peer review, and these perceptions were aligned with their practices. Nonetheless, the anonymized peer review process was also perceived as having its own weaknesses. For both anonymized and open peer review, the editors in chief identified several unique but connected problems, which we compiled into Table 3. In addition to these problems, five editors reported that peer reviewers were sometimes too hasty, providing little or no feedback to the authors. In these instances, the review reports were usually discarded and new reviewers invited.

**Table 3:** Problems of open and anonymous peer review identified by the editors in chief of the 12 Social Sciences and Humanities journals

PEER REVIEW	PROBLEM	EXAMPLES
**ANONYMIZED**	**Easy to abuse by editors**	*“I mean, obviously a position like mine can be abused in that way. Without question, it would be very easy to do so”* (E1) *“ultimately, the decision is mine”* (E4)
	**Difficult to credit reviewer**	*“one of the issues with blind peer review is, people never get credit for it. And increasingly, institutions are saying to faculty, tell us what you’re doing in your annual reviews. And we were getting requests to say, can you write a letter saying that I did the peer review”* (E6)
	**Facilitates unwanted gatekeeping**	*“reviewers riding hobby horses about their own views”* (E2) *“I’ve seen cases where it seems to me, even though you try to avoid it, that a reviewer has a disagreement or a grudge against something, just has a fixation on whatever. Has a indefensible opposition to something”* (E8)
	**Lack of reviewer accountability**	*“reviewer is grinding an axe behind the veil of anonymity”* (E8) *“the degree to which people have axes to grind or want to engage in some form of harassment or inappropriate behavior, a kind of single-sided process is probably not optimal at this point in time”* (E3) *
	**Enables bad language use**	*“review is shameful or aggressive or unprofessional or unethical”* (E5) *“the tone of the work has been more critical and constructive in a way that is not productive”* (E4) *“newly minted academics are sometimes a little too severe ... and too square occasionally as well”* (E11)
	**Difficult or impossible to carry out in practice**	*“Sometimes they say ‘I heard a paper at a conference two years ago and this looks like it’ and my response to that is: that’s fine”* (E8) *“I’m increasingly impatient with the norms for anonymizing, which almost becomes a game for reviewers to then try and figure out”* (E8)
	**Slows down communication**	*“Reviewer sends his/her comments to the editor who sends it over to the author who responds to the editor who decides whether s/he is able to evaluate or sends it back to the reviewer and then they send comments again. I don’t know if you could follow me”* (E12)
** OPEN**	**Institutional discredit**	*“that academic articles are double blind peer reviewed, it’s sort of taken often blindly as a gold standard”* (E11) *“how we can get the academy as a whole to value anything other than that kind of traditional double blind review?”* (E3)
	**Does not protect reviewers**	*“the standard reason is that the reviewer whose identity is protected has a license to be more candid”* (E2) *“I don’t know if it would be a very fair system to young researchers”* (E10)
	**Does not protect authors**	*“[anonymization] protects the reviewer and the author from any personal issue that might arise”* (E9) *“[only anonymity] will protect people from unfair biases by the reviewers”* (E1)
	**Reviewers cannot review candidly**	*“It has issues with what you dare to do as a reviewer”* (E1) *“to me the core is having it read by somebody who can be candid”* (E8)
	**Facilitates biases**	*“the prestige of the author might blind the reviewers, or the fact that you’ve never heard of the person”* (E8) *“it’s established as a research fact that* e.g. *women would have a greater chance of being published if they were going through a double blind peer review”* (E1)
	**Hinders finding reviewers**	*“it would even reduce the willingness of reviewers to participate”* (E12) *“it isn’t uncommon for me to go through maybe 12 declines before I find 2 reviewers. I’m not sure doing away with a blind review system’s the best”* (E5)
	**Editorial challenges**	*“somebody who’s writing about queer studies may say ‘I think that a true peer is somebody who is queer’ but I will not ask somebody what their sexual orientation is”* (E6)

* Referring to peer review where the reviewer can either guess or know the authors, i.e. including a degree of transparency

It is worth discussing a few of the 14 identified issues in more detail. First, we highlight the notion listed as “institutional discredit,” which some editors in chief considered a key barrier to even thinking about open peer review. Even though world-leading journals (such as *Nature*) support open review formats, many interviewees recognized double anonymized peer review as a default and felt they could not move away from it without sacrificing credibility. The pedigree of these standards appeared important particularly during the early years of the journals:

-
  “*We did not want to be innovative or be radical in any way. We wanted to have a journal that would be regarded and identified as a very standard traditional scientific scholarly journal.*” (E1)

Journals with a narrower regional or disciplinary focus likewise had a reason of their own for keeping the peer review process anonymous. With fewer authors and reviewers to draw on, anonymization was perceived as essential to reduce conflicts of interest when almost all experts are acquainted. One editor argued that, particularly in small, closely knit fields, peer-to-peer accountability based on disclosure of names might quickly devolve into “*interpersonal as well as disciplinary*” conflicts (E9). The same editor mentioned a frequent need to revise the language of reviewers, because their observations seemed addressed to the editors rather than the authors, and formulated in a “*language that would be shared among friends*,” i.e., not always respectful. “*So of course, I remove that – it’s unnecessary and insulting, and I rephrase it.*” Three other journals reported a similar policy, according to which a respectful tone was maintained by systematic review report editing.

For some editors in chief, the author’s identity was also relevant in the decision-making process. While the majority considered double anonymized peer review fair and objective, one editor felt that genuine fairness meant evaluating each submission (at all stages) in the context of the author’s career progress and background:

-
  “*I think it matters who the author is* … *We get senior scholars, well known people, and we get graduate students. And I think that work is going to be assessed in part in relationship to the identity of the author. And so I think it’s important for me, who’s going to be making some decisions about that, to know that. Now that then also means that I have to be conscious, as conscious as I can about my biases and so on, and I try to do that.*” (E2)

This was also the only journal in which open peer review practices were present. Its review process consisted of internal and external experts who would sometimes comment openly. At the other extreme, editors in chief considered transparency (i.e., knowing authors’ identities) unethical and pursued complete anonymity even when screening:

-
  “*We wanted to have a double-blind review process, because that is, as far as one can tell, the most fair way of selecting what gets published. When I screen a manuscript, I also have them screened without any information about the authors. There might be a surname attached, but I normally will not look up who that person is before I screen it.*” (E1)

Considering that all but one of the 12 journals were running double anonymous peer review processes, a common problem was a peer reviewer wishing to disclose their identity to the authors. When asked, three journals reported such instances to be somehow linked to the Peer Reviewers’ Openness Initiative [29]. The editors in chief handled these requests in opposing ways: either allowing the reviewers to sign reports or denying disclosure. One editor in chief felt that this transparency would challenge the agreed values, which involve respecting review anonymity:

-
  “*We’ve stated very clearly that our journal is a double-blind peer review journal. When they submit their work, that is the practice that we’re going to function under. I believe that if there is a desire on the part of the reviewer to want to make his or her name known to the author, that’s actually pushing on a value system that the author may not agree.*” (E4)

In line with the above, the conduct of a journal was systematically dependent on the context or conventions of the respective journal, suggesting that different journals (representing different subdisciplines) needed different approaches to reviewing.

-
  “*Ultimately we all want to publish the best of possible articles. So if one way works for an editorial board, fine. If another way works for a different journal, fine. In the end, people will read the final works published that contribute to scholarship. All the roads lead to Rome as far as I’m concerned.*” (E9)

One editor in chief, representing the humanities, explicitly called out the entire field as lagging behind and lacking proper peer review to begin with. According to them, “*there’s too little blind review or even peer review in humanities ... we’ve been leaning on this curatorial model way too much, which also gives editors way too much power – that’s something where we have a lot to learn from other fields*” (E1). Another editor in chief diagnosed open research practices as a reaction to bad research practices in *other* fields, and since “*we have not encountered that type of difficulty, we can go about having a real discussion about what are the upsides and downsides*” (E4). Meanwhile, one interviewee felt that openness, as such, was not considered relevant in SSH:

-
  “*Authors seem to have very little interest in open science. I’ve also spent some time for an open data initiative and I’m surprised – the extent to which I don’t see very many people actually interested. I just see a shrug of the shoulders. A kind of ‘Eh, this doesn’t really apply to us, why would I want to do this? It’s just more work, it’s more effort.*’” (E3)

Every journal, except one, supported publication formats such as book and narrative reviews or critical commentaries, which were not peer reviewed or were peer reviewed differently. Lacking standard review processes, they might not play a role in promotion or hiring, especially for early career researchers:

-
  “*To create something that isn’t going to provide people with a line they can put on their CV, under peer reviewed journal publication, that’s a very tough thing to ask of people. And this is, to me, an incredible frustration because doing things … that would be valuable contributions to the scholarly conversation are not going to count.*” (E3)

The above institutional reality – only traditionally peer reviewed original articles count toward people’s career progress – also served as an incentive for the editors to direct their own resources of time and innovation towards manuscripts following traditional peer review.

### How do the editors in chief position their journals’ review and publication practice between policy, ethics, and pragmatism?

All the interviewees’ review processes involved considerations of current policies, ethical challenges, or pragmatic issues. The most fundamental point of agreement among the editors was a unanimous satisfaction with the volunteer work of their peer reviewers, who represented the pragmatic cornerstone of running an academic journal. Three journals estimated the prevalence of “bad” review reports numerically, claiming them to be 1–2% of all received reports. To maintain high quality in their review processes and make sure that the review system would work in the future as well, the interviewees disclosed systematic and non-systematic means by which they keep track of both internal and external reviewers.

-
  “*We actually have this internal system, and most of us remember to rate the reviewer. When you go out and look for a reviewer, if you look in our system, you would see if one of the other editors had rated the reviewer very low.*” (E10).

The concept of quality in the above and other cases was rarely a matter of content alone, but also reliability and speed. Reviewers who did not respect deadlines or were difficult to communicate with could likewise be classified bad quality, even if their feedback was appropriate.

Related to the transparency of the review process, the interviewees listed miscellaneous elements that they considered topical. For instance, one editor in chief talked about marking submission, revision, and acceptance dates in the final article as a feature that can remove doubts about the process, however, it may also turn against the journal:

-
  “*I see on some journals now the notation ‘manuscript submitted on such and such a date, accepted such and such a date, published such and such a date.’ I don’t think that’s a very revealing statistic or data point for a journal like ours where, in my opinion, so much of that timeline is outside my control. But it’s part of greater transparency.*” (E6)

None of the journals provided financial compensation for their external peer reviewers. Finding external reviewers to work for free was one of the core challenges for journal editing, as multiple editors noted how it would not be unusual to ask up to 15 people to review before finding two who would agree. The trend was occasionally described as increasing (“*there’s a momentum building up*,” E5), in which case the editors in chief felt unequipped to solve the problem due to lacking means for compensating the review work that they still needed to run the journal: “*How do you reward reviewers? Because this whole gift economy depends on reviewers’ unpaid labor*” (E8). Systems like Publons were mentioned as possible solutions, with the caveat that they would not remove the original problem of volunteer work.

The represented journals actively pursued editorial diversity, for instance, by carefully managing the board with ethnicity, gender, and regions in mind. Nonetheless, two editors in chief expressed surprise that such diversity should even be considered. In the external review process, the defining diversity concerns were about disciplinary or methodological domains, i.e., having both sides of the coin would benefit the review, especially in polarized topics.

A further complicating matter of policy and practice were journal metrics, which for some served to self-assess their own performance (also by funding institutions), while at the other end of the spectrum, such numerical values were considered flawed and irrelevant. Half of the editors in chief indicated interest in the statistics, usually provided by the publisher. Only those whose journals that were up for review through publishers felt that clicks, subscriptions, and other metrics mattered in practice. When asked specifically about impact factor, replies ranged from moderate interest (“*it’s important for the publisher for sure—but it’s also important for us*,” E12) to complete defiance (“*fuck the impact factor*,” E3).

Although all editors in chief, except one, professed to be aware of the impact factor among similar metrics, there were no attempts at influencing journal policy from publishers or affiliated academic associations. On the other hand, the editors generally admitted being pleased with their journal’s success, and since this success was typically validated by high journal rankings and peer recognition within the field, some felt that careful self-reflection was needed when assessing potential “high impact” manuscripts.

-
  “*The thing goes back to the idea of rankings and stuff. So maybe I should publish more canonical stuff if I want to get higher. I don’t want to think that way, but I know that [reality]. So how is that going to affect my practice?*” (E2)

Again, the question was conventionally tied to the reality of academic careers and work. Even if the editors did not consider metrics relevant, many of their submitters did. In this way, the metrics had an impact on the journal’s profile and prestige, and whenever such metrics were not disclosed, the editors could receive requests to make them transparent.

-
  “*We occasionally get a request from an author for what our journal impact factor is. That typically comes up when an author is up for review, promotion, or tenure. And I’ve written letters back to them saying it’s not our job to participate in the tenure review.*” (E6)

In sum, the editors’ personal viewpoints regarding the review process, its management, and related journal metrics were occasionally in conflict with their ethics as well as their journal’s practice. By and large, the editors in chief were aware of this and often actively pursued solutions, which nonetheless were difficult to implement due to the institutional policies across academic systems and fields.

### How do the editors in chief situate their own powerful role in the manuscript review process and how is that role negotiating open science principles of the review process?

The interviewed editors in chief were widely aware of their decision-making power, but as found in previous research [19], they did not consider it problematic. Although the editors in chief were generally satisfied with the current anonymized peer review and were not planning to change it soon, other open science practices like inviting authors to share their data and making paywalled issues open access were mentioned [30, 31].

**Fig. 1:**
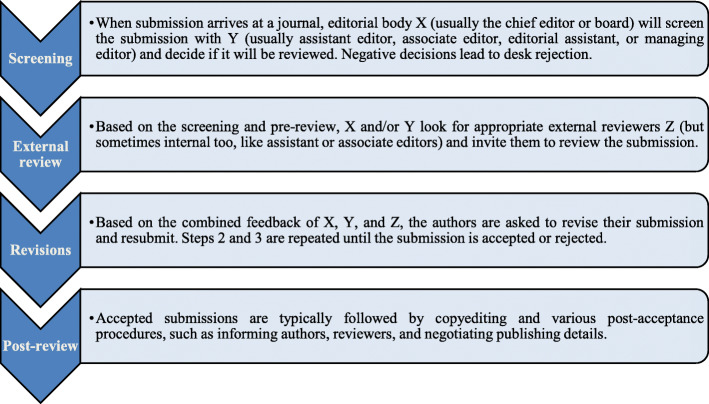
The four stages of the manuscript review process by the 12 Social Sciences and Humanities journals whose editors in chief were interviewed for this study

All interviewed editors in chief employed multiple stages for reviewing submissions, and these stages largely followed the model presented by Horbach and Halffman [32] with minor exceptions. In journals that were independent or affiliated to smaller publishers, the editors in chief (and other editors) often had several areas of responsibility, i.e., were intensively involved in all stages, and sometimes served as “external” reviewers (in such cases different editors would usually handle the manuscript, allowing the reviewing editors to be anonymized) (Fig.1).

The most critical editorial decisions take place at the very beginning of the peer review process, as desk acceptances and rejections are made without external reviewers. Five unique reasons were identified as warranting a desk rejection: *lack of fit with journal scope*, *poor overall quality*, *ignoring relevant literature*, *narrowness or low impact*, and instances where *too many similar manuscripts were already in review or published*. In one exceptional case, the editor in chief noted almost all desk rejections to derive from the fact that the journal’s name was similar to other journals with a different profile, which made authors systematically submit manuscripts that address out-of-scope topics.

Anonymity at the desk stage was considered both impossible and impractical. One interviewee, for instance, was ready to let assistants make desk decisions, but in a way that would allow the editor in chief to supervise the process in an open format:

-
  “*That has to be open. For example, you would never assign a reviewer who’s in the same department as the author. So you need to know where the author is. You also want to avoid assigning a reviewer who was likely to have been the author’s advisor. So that really can’t be blind, or you’ll end up just sending things to coauthors or work colleagues. I am much less concerned with the anonymity of authors than the anonymity of reviewers.*” (E8)

One of the smaller journals had a system that inverses the dynamic of the screening stage: a significant proportion of submissions are first informally suggested to the editor in chief, who then seeks input from experts in the field to support authors in developing proposals. Only then proposals are submitted and subjected to a double anonymized peer review, which then has a very high acceptance rate. To this editor in chief, nurturing promising submissions from the start strengthens the quality of the journal while guaranteeing a steady stream of high-quality publications – a desirable strategy because “*we are in the business of publishing, not punishing*” (E9). This feed-forward form of curation creates less need for critical feedback (and rejection) in the later stages: “*I like to think that we do a good job prior to the submission, so the author can confidently send an article and receive a positive, constructive feedback*” (E9). The described process reminds one of the “registered report” article format [33], which was not explicitly used by any of the 12 journals.

In some journals, editorial power was coordinated by having the screening supplemented with a full editorial review. In these cases, one or more persons of the editorial staff read the entire manuscript and provided (signed or anonymous) feedback before potentially moving to external review:

-
  “*Sometimes we get articles from [country] that are 1.5 page – then it's a desk rejection. But if it's a full article, the editorial manager will assign it to one of the other editors. And then it's the editor's task to go through the article.*” (E10)
-
  “*Chief editors have a look at the first round, we can then give a desk reject right away. But if it goes further, then two members of the editorial board read the text and assess whether it's good to go to review or not. We might not desk reject, but send ‘OK, didn’t go to review yet, but if you do these changes, we’ll reconsider.’*” (E7)

In the external review phase, editorial power is not direct but manifests in reviewer selection. This could be done by the editor in chief or another staff member, or as a collective effort. Higher volume journals had reviewer databases with hundreds of potential experts; smaller journals would mainly recruit via the personal networks of the editorial staff. All editors agreed that academic qualifications were the primary selector, supported by a whole range of other criteria.

-
  “*It’s because they’re considered experts. It’s because sometimes we know them personally. It is because they are more committed, because they’re on the board. And it is also because they are familiar with the journal, the direction of the journal, the expectations and the level of quality of the journal.*” (E5)

Altogether, nine unique criteria were considered relevant in choosing the reviewers: *age*, *biases*, *commitment*, *political/theoretical position*, *distance*, *expertise*, *nationality*, *personal preferences*, and *recommendations*. Additionally, two journals were proud to have “harsh” or “super” reviewers who could be used for the most challenging tasks:

-
  “*And then there are my sort of super reviewers. They are people whom I have just learned to trust. Who will first of all do it if they say they’ll do it, but also are good at sort of sifting through, reading and picking things up. These are often people who have been journal editors or just have a track record. And if I had a structure of associate editors, these are people who would be associate editors.*” (E5).

The role of external review was somewhat polarized among the journals. On one side, certain editors consider the external review to be advisory instead of decision-making. These editors define their roles rather as curatorial or akin to editors in book publishing. Beyond assuring high quality publications, they see their responsibility in stimulating innovative impulses and helping authors bring their concepts to full fruition in a collaborative process.

-
  “*we don’t follow the peer review slavishly, but then again, the issue of not recognizing what the reviewer was saying has not really arisen – we end up reading every single piece submitted and everything, and then every piece that is published in the journals, we go over each one of us, more than once.*” (E11)

At the other extreme, some editors in chief were utterly clear about operating as nothing but mediators between reviewers and the reviewed submissions. These editors pursued first and foremost the assurance of scientific quality, and in this picture, the external reviewers served as “objective” measures.

-
  “*I tend to view myself as an umpire. I’m not qualified to make these decisions. My job really is to try and ensure that the reviewers are appropriate and that the reviews are fair, to the extent that I can. And I’m sure there are mistakes.*” (E8).

It is worth re-noting that sometimes editorial review was merged with the external review. In these scenarios, internal editorial reports could be delivered based on either double or single anonymized principles. None of the editors in chief expressed concerns or policies regarding the disclosure of these internal processes, but we also did not inquire directly about them.

-
  “*Sometimes it gets complicated and then the person I ask will be somebody on our editorial board who has been fairly helpful in the past, because you're asking them basically to follow through the entire editorial history of this. I'll send them the whole thing, like, ‘here's the history, here are the reviews, what do you think.’*” (E8)

Despite collectively following and agreeing on the previously discussed benefits of anonymized external review, doubts were voiced regarding anonymized communication in the revision phase. One editor in chief felt that the initial anonymized processes would benefit from increased transparency after the necessary “gatekeeping” had been cleared out:

-
  “*often what’s far more useful is a sort of semi-collaborative editorial process that follows after double blind peer review. That’s where the improvements are really made. This is just a kind of initial gatekeeping, and sometimes it’s useful and sometimes tokenistic.*” (E5)

In cases such as the above where editors expressed a personal liking for opening the revision process, they also cited institutional requirements that would not index their journal as a proper scientific journal without anonymity involved. For instance:

-
  “*A completely open process, I think, is far more plausible. But then the issue is also that I’ve been on review committees where people have said, well, if this has not gone through a double blind review, it doesn’t count as much. So I still think there’s a huge hurdle to overcome in terms of how we can get the academy as a whole to value anything other than that kind of traditional double blind review.*” (E3)

In the post-review phase, most journals draw strongly on their editorial assistants for technical quality assurance such as checking the integrity and completeness of the citations. In this phase, pragmatic differences, primarily relating to the ways in which the journals are financed, emerge. The journals that are primarily or exclusively financed by universities report dwindling subsidies, whereas journals with strong ties to associations or publishers appeared more stable. Relations to publishing houses were characterized unanimously as harmonious and unproblematic, except for some unease in cases of journals facing an upcoming periodic review of viability.

Only one journal employed technical means to assure quality control beyond peer review, as they had recently started using plagiarism detection software (“*now we run all papers through a system to see a possible relapse*,” E12).

Transparency-wise, the foremost question at this phase was whether author or reviewer identities could be opened after a positive publication decision.

-
  “*When I send an acceptance notice and say ‘dear so-and-so we’ve accepted your article’ I send that to the reviewers as well. It seems to me at that point I can include the name of the author. I mean, we’ve made the decision. But sort of automatically I take it out. But I keep thinking, why am I taking out the name of the author?*” (E8)

Finally, some editors in chief perceived the articles that they publish in the larger continuum of scientific evolution. Namely, the peer review of a publication is not something that takes place in or by the journal alone; rather, journal review is one evaluative event in an article’s life, which continues post-publication as peers read and review it in academic forums:

-
  “*So if the paper is not good, it will be lost in history, it won’t get citations. If it’s really influential but problematic, there will be some dialogue, there will be some criticism, there will be some contrasting results presented and so on.*” (E5)

From the above point of view, editorial power merges with the larger system of research evaluation that has already started before submission and continues after publication.

## Discussion

In this study, we were interested in how open peer review is perceived by respectable SSH journals and how their own review processes align with those perceptions. All but one of the journals applied complete double anonymized peer review. The interviewees voiced that open peer review processes are perceived as less reliable and less fair than anonymized peer review processes, primarily due to the lack of protection the former provide for both authors and reviewers (Table 3). However, as editors in chief, they felt that having access to authors’ names at the submission stage was essential, for instance, to being able to select appropriate reviewers. Following the above, they generally considered double anonymized peer review to be the “gold standard” in terms of ethics and practice and felt that switching to open peer review could also lead to losing institutional support and academic credibility. It is possible that in the SSH domain, anonymity in peer review is perceived to be more important than in other fields. Given the increasing calls for stricter fact-checking in academic publications [34], questions of quality assurance were a key area highlighted in the interviews.

We also explored how the editors in chief position their journal’s review and publication practice between policy, ethics, and pragmatism. The interviewees systematically spoke of their journals’ (double anonymous) peer review processes to be largely defined by academic policies and ethical as well as pragmatic challenges. At the same time, the practices of journal peer review, reviewer selection, and editorial oversight were contextualized as a part of an overarching system. In this system, studies will have been vetted by university ethics boards and national or international grant givers. The journal then assesses through peer review the relevance and innovation of the research, while at the same time itself being subjected to oversight by a publisher or other funder, mostly based on success criteria like the impact factor and number of downloads. We acknowledge that emerging guidelines like Plan-S and best-practice recommendations from societies such as the Committee on Publishing Ethics (COPE) further complicate the relationships between authors, funders, and journals, among others. At the same time, the editors in chief expressed satisfaction with the means and the degree of quality, particularly in their own publication. One journal had even begun using a software tool to verify integrity and originality; however, the volunteer work of the external peer reviewers was perceived as an unchanging pragmatic cornerstone in all journals. In the end, our study witnessed a view from which editorial work and peer review serve not merely research publishing, but a process elevating the quality of said research.

Finally, we were interested how editors situated their own powerful role in the review process and how in that role they negotiated open science principles in it. We did not find the journals explicitly regulating editorial power, but the editors voiced awareness of it and recounted various implicit strategies dealing with the issue. The pragmatics of organizing scientific journal peer review remain difficult to completely align with the professional ethics generally striven for – “papers do go to referees, but then editors choose referees” [35]. The issue itself was perceived universal. Some editors in chief moderated their power through self-policies, along the lines of publishing articles they disagree with but find important, whereas others distributed editorial responsibilities across a large editorial team. Some of the interviewees were the founding members of their journal and perceived a strong identity between themselves and the journal. In only two cases interviewees reported fixed chief editorial tenures, whereas five had been in their function for one or more decades. Journals with large editorial boards and shared editorial duties may have further fine-tuned means for power management that our interviews were not able to chart; future research may shed more light on those. We must stress, however, that some of the editors in chief were in the process of considering new open science practices (like requiring data sharing) for their journals – and if they were motivated to move toward more open peer review processes, there seem to be no pragmatic limitations for doing so.

All of our interviewees stressed the intricacy of the process of selecting, editing, and accepting/rejecting submissions by the profile of the journal. Although these procedures are explicitly stated on the websites of all journals as a matter of course, the subtleties of the selection processes that inform the all-important early stages of screening as well as editorial review were difficult to articulate even for the seasoned editors we spoke to, and were not easily formalized in written, generalizable form. This evidences how review criteria, such as the “big four” listed in *Responsible Journals* [36] – rigor, impact, novelty, and fit – were not easily measured and, in the end, largely depend on the editor’s or reviewer’s subjective position. In this context, the transparencies related to *who* decides and *how* are central, as the criteria per se may operate as guidelines for interpretation. Following the above, a key outcome of our study is a brief policy recommendation regarding peer review transparency beyond reporting the “open” or “anonymous” types of the external experts’ reports – which is only a small part of any review process. Instead of disclosing “at what stage of the publication process does review take place” [36], journals should communicate the transparency elements of their peer review by listing which stakeholders contributed to the decision *stage by stage*, and especially if the process is “double anonymous” (Table 4).

**Table 4:** A suggested contributor list for journal decision making in scientific manuscript peer review. Note that contributors may also be listed anonymously

STAGE	Executive decision	Assisting in decision	Consulted for decision
**Screening**	E.g., editor in chief or associate editor(s).	E.g., editor in chief or associate editor(s).	External or internal experts.
**External review**	E.g., editor in chief or associate editor(s).	E.g., editor in chief or associate editor(s).	External or internal experts.
**Revisions**	E.g., editor in chief or associate editor(s).	E.g., editor in chief or associate editor(s).	External or internal experts.
**Final decision**	E.g., editor in chief or associate editor(s).	E.g., editor in chief or associate editor(s).	External or internal experts.

### Limitations

As a qualitative investigation, our study was limited to a small and non-representative sample of editors in chief from SSH disciplines – and most notably, we did not interview any journals using open peer review alone (one journal selectively mixing anonymized and open). Although this must be added as a limitation, we may recall a previous review [2] finding only *one* humanities journal using open peer review, while Scimago alone indexes nearly 500 humanities journals. This considered, our participants might represent the reality rather than selection bias.

In future research, the presented findings could be utilized as hypotheses and tested quantitatively with a larger and more representative sample of editors. On the other hand, self-reported practices, as in this study or survey research, are never direct evidence for actual practices – future studies should also keep assessing journal practices by other methodologies. We also did not interview editors in chief outside the SSH, so we do not know if the views presented here are unique to the SSH or if editors in chief in other fields share these views. Finally, we were not able to participate in data sharing due to some of the interviewees requesting anonymity; nevertheless, at least a few of our interviewees would have been willing to share their interviews openly even without editing.

## Conclusions

The scientific manuscript review process consists of multiple stages in academic journals. Our study suggests that, in the Social Sciences and Humanities, carrying out the stage(s) of external review by the double anonymized principle is perceived generally as a “gold standard” and superior to its open alternatives for various ethical, practical, and policy-related reasons. As for this *and* other stages of review, we recommend journals to communicate the transparency elements of their processes by listing all stakeholders involved in reviewing and decision making. To further improve their transparency, journals could describe their process in the *Responsible Journals* platform [36].

## Supplementary Information

**Additional file 1.**

## Data Availability

The data will remain unshared due to the requests of some interviewees. Parts of the data may be shared by request to the authors.
