# Supplementary material for: Transparency of peer review: a semi-structured interview study with chief editors from social sciences and humanities
Source: Res Integr Peer Rev. 2021 Nov 18;6:13. doi: 10.1186/s41073-021-00116-4 (PMC8598274; doi:10.1186/s41073-021-00116-4)
Supplement: Supplementary file 1 — Additional file 1. [file 41073_2021_116_MOESM1_ESM.docx]

**Table of Contents**

1. Further literature on peer review
2. Interview
3. **Further literature on peer review**

Although almost all previous studies (as well as our interviewees) refer to “closed” peer review processes as “blind” or “double blind,” in this article we use the term “anonymized peer review” following Jones and others [1]. Ford’s [2] list of eight open peer review formats, based on 35 earlier studies, provides one more illustration.

*Signed review* reveals the identity of the reviewer to the author. In *disclosed review* both the author and the reviewer are identified to each other. *Editor-mediated review* reveals the editor(s) as a decision-making or reviewing entity for the author. *Transparent* *review* occurs in a public (typically online) environment with authors, reviewers, and editors all disclosed. *Crowd-sourced* *review* additionally allows community members to participate in the public review. *Pre-publication* *review* takes place during an extra preliminary review stage, as a work is evaluated in an open environment (e.g. preprint server) before entering the actual publication process. *Post-publication* *review* enables direct public commenting and criticism of a work after its publication on the journal platform. *Synchronous* review enables a work to be published in a dynamic format with continuous public review and editing.

Ford’s [2] list is not exhaustive and lacks, for instance, one relatively common form of open review: a single blind review where the authors’ identities are revealed to the reviewer, but not the other way around. Regardless, the above suffices to demonstrate that the notion of “open” is multifaceted and complex, and that there are several dimensions of openness that all potentially influence the review process in diverse ways. These potential influences have been studied respectively in detail too.

In a large-scale experiment carried out by Walsh and colleagues [3] with the *British Journal of Psychiatry*, the scholars asked the peer reviewers of 408 manuscripts to sign their review reports and disclose their identities to the authors. A total of 76% agreed to sign, and their review reports were found to be of higher quality, more courteous, took longer to complete – and they were more likely to recommend publication. The scholars conclude:

*time commitment involved in reviewing might be too arduous for referees if the peer review process were opened up, especially when one considers the increased workload resulting from the loss of reviewers who refuse to sign their names. Although signing appears to make reviewers more likely to recommend publication and less likely to recommend rejection of papers, it is important to remember the role of the Editor in this process [moreover] junior reviewers may hinder their career prospects by criticising the work of powerful senior colleagues* (Walsh et al. 2000) [3].

While some studies have also reported less clear or no differences between signing and non-singing reviews [**Error! Reference source not found.**, 5], the current consensus points at the exact benefits and deficits originally indicated by Walsh’s group [**Error! Reference source not found.**, 7]. These open review findings have been studied further still, with various sub focuses such as reviewer bias.

Manchikanti and colleagues [8] cite evidence for at least six unique biases that have been found to corrupt the peer review process: confirmation bias (supporting existing beliefs), conservative bias (resistance against new methods/theories), bias against interdisciplinary research (applying single discipline criteria to multidisciplinary studies), publication bias (preference for positive results), bias of conflicts of interest (judgment based on personal benefits), and content-based bias (numerous biases produced by a subjective position – including “ego bias” that makes reviewers favorable for work that cites the reviewer). The scholars entertain transparency via open peer review formats as an antidote to these issues; however, they also repeat the previously noted challenges that come along with open review: increased difficulties related to recruiting reviewers and having honest criticism. Moreover, in the case of public open review, the review process can be complicated with commentators or critics who may not qualify as experts.

In summary, the academic world – represented by 28,094 scientific journals that publish some 2 million scientific articles annually [9] – has come to employ multiple diverse review strategies in their publication processes to assure quality and fight biases. These peer review strategies have several transparency elements that surface at different stages of the publication process. A near consensus is that fully anonymous peer review practices are good for providing reviewers a safe space to criticize candidly, which also facilitates the speed of the review process. Fully open review processes, in turn, are generally good at motivating the reviewers and holding them accountable, thus increasing the overall quality of feedback and making biases as well as conflicts of interest visible.

In comparison to the relatively recent emergence of organized open science approaches, the discussion and study of scientific peer review (sometimes referred to as ‘journalology’) is old and widespread [10, 11]. Already in the early 2000s, Garfield [12] found no less than 3.720 publications focused on the peer review process alone. Such a number of studies naturally covers a great range of subtopics, but the key question remains unsolved to date [13]: how to verify scientific quality in a way that is ethical, reliable, and possible to carry out in practice?

References

1 Jones L, van Rossum J, Mehmani B, Black C, Kowalczuk M, Alam S, Moylan E, Stein G, Larkin A. A Standard Taxonomy for Peer Review. OSF; 2021 [Cited April 15, 2021] Available from osf.io/68rnz

2 Ford E. Defining and characterizing open peer review: A review of the literature. Journal of Scholarly Publishing. 2013;44(4):311–26.

3 Walsh E, Rooney M, Appleby L, Wilkinson G. Open peer review: a randomised controlled trial. The British Journal of Psychiatry. 2000;176(1):47–51.

4 Godlee F, Gale CR, Martyn CN. Effect on the quality of peer review of blinding reviewers and asking them to sign their reports: a randomized controlled trial. JAMA. 1998;280(3), 237–40.

5 Van Rooyen S, Godlee F, Evans S, Black N, Smith R. Effect of open peer review on quality of reviews and on reviewers’ recommendations: a randomised trial. BMJ. 1999;318(7175), 23–7.

6 Shanahan DR, Olsen BR. Opening peer-review: the democracy of science. J Negat Results Biomed. 2014;13(2). <https://doi.org/10.1186/1477-5751-13-2>.

7 Moylan EC, Harold S, O’Neill C, Kowalczuk MK. Open, single-blind, double-blind: which peer review process do you prefer? BMC Pharmacol Toxicol. 2014;15(55). https://doi.org/10.1186/2050-6511-15-55.

8 Manchikanti L, Kaye AD, Boswell MV, Hirsch JA. Medical journal peer review: Process and bias. Pain Physician. 2015;18(1), E1.

9 Ware M, Mabe M. The STM report: An overview of scientific and scholarly journal publishing. Fourth Edition. 2015. Available from: https://www.stm-assoc.org/2015_02_20_STM_Report_2015.pdf.

10 Burnham JC. The Evolution of Editorial Peer Review. JAMA. 1990;263(10):1323.

11 Bornmann L. Scientiﬁc peer review: An analysis of the peer review process from the perspective of sociology of science theories. Human Architecture: Journal of the Sociology of Self-Knowledge. 2008;6(2):3.

12 Garfield E. Historiographic mapping of knowledge domains literature. Journal of Information Science. 2004;30(2):119–45.

13 London B. Reviewing Peer Review. *J Am Heart Assoc.* 2021;10:e021475. doi: 10.1161/JAHA.121.021475

1. **Interview**

Q0: Introduction and description of Journal focus

- [We brief participants on their consent and our storage of data and processing].
- How is your journal published (i.e. open access, online only; optional print publication for members/paying customers, print-on-demand) and in what way is the journal presented online (HTML or PDF? With DOI?)?
- How long have you been editing the journal?
- Is your journal independent or part of an organization or association and how is your journal financed?
- Do you consider your journal to be mono- or multidisciplinary? If monodisciplinary, what discipline do you identify with? How does this vision of the journal influence your review and publication practices?

RQ1: In what way do highly ranked humanities and social science journals perceive of open peer review processes and how do these perceptions materialize in the actual processes they employ?

- Does your journal adhere to open science principles? If so, how strictly and in which respects (i.e. peer-review, open data, pre-registration, etc.)? Does the fact that some manuscripts are stored publicly before peer review have an impact on the review process?
- Does your journal practice a blind or open review process? What are the reasons for the review process?
- How do you understand the concepts “open” and “closed” review? (There are many forms, see e.g. Ford 2013. Are editors familiar with these diverse forms?)
- How much have you recently engaged with journalology and/or are you aware of the current state of research or discussions considering review practices?
- If your journal is interdisciplinary, how do you take this factor into consideration in the peer review process?

RQ2: How do the editors in chief position their journal’s review and publication practice between policy, ethics, and pragmatism?

- How do you instruct reviewers on the proper review practices? Are the criteria predominantly geared toward knowledge dissemination, impactful research, or other societal values?
- How collaborative is the review process? Are reviewers and authors engaging in a direct dialogue to improve the final paper, or are they expert evaluators for the editors’ decision making process? Are there additional actors involved (e.g. non-blinded commentators)?
- Are you familiar with the peer reviewers’ openness initiative, and what are your experiences of it in your journal?
- How do you track review/er quality, and have you observed any common factors for good/bad reviewers? (Evidence shows that older reviewers are clearly worse.)
- What are the other means to detect errors in studies, except for peer review? (Evidence shows that “peer review doesn’t work”.)

RQ3: How do the editors in chief situate their own powerful role in the peer review process and in what way is that role negotiating open science principles of the peer review process?

- How are reviewers selected? (Evidence shows that conflicting reviews is a good sign. Agreeing reviews is a sign that review process does not work. ”Reviewers should be selected precisely because of their different perspectives, judgement criteria, and so on (Stricker 1991).” [It is unlikely that one paper can meet opposing criteria.]
- Do you publish statistics about your journal openly? If yes, what kind of and why (etc), and if no, why not?
- What role do editors play in the process of deciding on publication? (How large is the number of desk rejections? How binding are the peer review results, i.e. do you ever accept papers recommended for rejection or the other way around?)
- Publication is not only about the quality of work but also about its impact/relevance -- how do you define and measure this factor on desk and external evaluation? Pre-screening procedures obviously have a strong potential bias.
- What are you comfortable with, in terms of storing this interview -- audio, text, anonymous? Can we refer to you and your journal by name in the publication to contextualize your responses? Can we disclose your journal’s participation in the study while anonymizing individual replies?
